# Evaluation of serum samples in long cryopreservation for SomaScan proteomics and sex differences in elderly Japanese adults

**DOI:** 10.1038/s41598-025-14464-4

**Published:** 2025-08-14

**Authors:** Ruriha Beppo, Yuki Ohashi, Ken Yamamoto, Fumie Kinoshita, Tomoko S. Kato, Masahisa Katsuno, Tatsuaki Matsubara, Mitsuhiro Yokota, Sahoko Ichihara, Masahiro Nakatochi

**Affiliations:** 1 Department of Nursing, Nagoya University School of Health Sciences, Nagoya, Japan; 2 Public Health Informatics Unit, Department of Integrated Health Sciences, Nagoya University Graduate School of Medicine, 1-1-20 Daiko-Minami, Higashi-ku, Nagoya, 461-8673 Japan; 3 Department of Medical Biochemistry, Kurume University School of Medicine, Kurume, Japan; 4 Department of Advanced Medicine, Nagoya University Hospital, Nagoya, Japan; 5 Division of Cardio-Vascular Medicine, Department of Internal Medicine, Kurume University School of Medicine, Kurume, Japan; 6 Department of Neurology, Nagoya University Graduate School of Medicine, Nagoya, Japan; 7 Department of Clinical Research Education, Nagoya University Graduate School of Medicine, Nagoya, Japan; 8 Faculty of Human Sciences, Aichi Mizuho College, Nagoya, Japan; 9 Kurume University School of Medicine, Kurume, Japan; 10 Department of Environmental and Preventive Medicine, Jichi Medical University School of Medicine, 3311-1 Yakushiji, Shimotsuke, Tochigi 329-0498 Japan

**Keywords:** SomaScan, Proteomics, Gonadotropin, Adiponectin, Resistin, Proteomic analysis, Biomarkers

## Abstract

Many biobanks store biological samples and use them for various analyses, including proteomics. Therefore, it is necessary to consider the denaturation of target proteins during long-term storage. We analyzed 16-year-old cryopreserved serum samples using the SomaScan platform, a novel proteomic assay, to determine whether adiponectin and resistin concentrations were consistent with those measured in our previous studies using a different platform. The results suggested that long-term cryopreserved serum samples could be used for future studies of at least adiponectin and resistin, which are closely related to the pathophysiology of diabetes, cardiovascular disease, and other metabolic diseases. Therefore, 7,289 SomaScan-assayed circulating proteins were compared between 20 men and 20 women aged ≥ 50 to determine sex differences. In total, 20 serum proteins showed significant sex differences. Of these, proteins that showed a more than two-fold difference in concentration between sexes contained heterodimeric forms of gonadotropic proteins such as CGA|FSHB, CGA|CGB3|CGB7, and CGA|LHB, which are the biologically active forms of these hormones. The present study is the first to report the possibility of using long-term cryopreserved serum samples for the SomaScan assay, and the results show that the SomaScan assay may be useful for analyzing sex differences focusing on gonadotropic hormones.

**Supplementary Information:**

The online version contains supplementary material available at 10.1038/s41598-025-14464-4.

## Introduction

Biological sex differences influence the signs and symptoms of age-related diseases in addition to inherent physical features. For example, the incidence of ischemic heart disease, the leading cause of death, increases rapidly in women after menopause^1,2^. In addition, women have higher rates of atypical angina symptoms (e.g., jaw pain and nausea) than men^2–4^and this potential diagnostic gap caused by sex differences may be contributing to increased mortality among women due to cardiovascular disease^5^. Similarly, there are biological sex differences in both the prevalence and symptom progression rate of Alzheimer’s disease^6,7^. Although the increasing life expectancy of women is considered a major reason for the high prevalence of Alzheimer’s disease, endocrinological changes associated with aging and lifestyle are a contributing factor^8–11^. Furthermore, there are several disparities in the aging trajectory between men and women^12^. Therefore, when considering sex differences in pathophysiology, it is imperative to understand how biological sex has an underlying effect at the foundation of disease onset during aging.

Almost all body cells are in contact with the blood, and many of them release some of their contents into the bloodstream through exocytosis or cell death (e.g., necrosis and apoptosis). Therefore, circulating proteins are recognized as potential biomarkers, and their fluctuations reflect physiological dynamics, aging, and organ dysfunction^13^. Proteomics has evolved dramatically in recent decades through the development of bioinformatics and innovations in measurement technology. To fundamentally understand biological systems, it is important to measure blood proteins over a dynamic range employing the greatest quantitative precision possible. In addition to liquid chromatography-mass spectrometry^14–16^affinity-based measurement technology is the most common method used to measure blood proteins, and it is becoming widely used. However, the SomaScan assay is a multiplex affinity-based proteomics platform that partially overcomes limitations, such as the dynamic range, of conventional methods using nucleic acid binders^17,18^. Indeed, a recent large-scale study using the SomaScan assay revealed novel age-related signatures and pathways that were not previously anticipated^19^ between men and women, and the results provided deeper insights into age-related fluctuations in plasma protein molecules caused by sex differences. However, this large-scale study consisted of Europeans and Americans^19^and the proteomic signature of biological sex differences in Asian ethnicities remains unclear. Therefore, we aimed to identify circulating protein concentrations in men and women of Asian ethnicities.

As blood samples are one of the easiest biological samples that can be collected, many biobanks store blood samples for a wide range of uses, including proteome analyses^20,21^. However, although these are important resources for bioinformatics, the storage period may potentially affect measurement results^20–22^and the effects of the storage period have not been adequately investigated using the SomaScan assay compared to those using conventional proteomics platforms. We previously measured serum adiponectin and plasma resistin concentrations through enzyme-linked immunosorbent assay (ELISA), a conventional immunoassay method^23,24^and measurements of these proteins using ELISA and SomaScan assay have been found to match with high accuracy^25^. These available ELISA-measured data prior to long-term cryopreservation prompted us to evaluate whether these stored serum samples could be reliably used with the SomaScan assay, a novel proteomics platform. Therefore, in addition to identifying circulating protein concentrations in men and women of Asian ethnicities, we also evaluated the use of long-term cryopreserved samples in the SomaScan assay.

## Methods

### Study design and participants

This cross-sectional observation study was designed as a part of the Kita-Nagoya Genomic Epidemiology (KING) study [ClinicalTrials.gov identifier: NCT00262691]^23,24,26,27^. The candidates were 1,876 individuals (887 men and 989 women) who participated in the KING study in 2007 and whose circulating adiponectin and resistin concentrations were measured in our previous studies^23,24^ using a latex turbidometric immunoassay (Otsuka Pharmaceutical Corporation, Osaka, Japan)^23^ and ELISA kit (LINCO Research, St Charles, MS, USA)^24^respectively. We matched men and women by age completely, and a total of 40 Japanese participants (20 men and 20 women) were eventually selected as the study participants. All 40 participants were ≥ 50 years old, and the mean age (standard deviation [SD]) was 64.9 (6.4) years old.

All serum samples used in this study had been stored at -80℃ since centrifuging whole blood from the KING study participants in 2007 (approximately 16-years). These whole blood samples were collected from the veins of participants who had fasted overnight. In the present study, the stored serum samples of the selected 40 participants were used for proteome analysis. As noted above, those serum samples were thawed and refrozen only once for adiponectin measurement.

### Serum proteomics measurements

We used the SomaScan Discovery platform V4.1 (SomaLogic Inc., Boulder, CO, USA), which is based on slow off-rate modified single-stranded DNA aptamers (SOMAmers) binding to specific target proteins. SOMAmers exhibit high specificity and affinity for proteins and minimize nonspecific binding interactions because of their slow dissociation rates. Previous studies have reported high assay reproducibility and low technical variability with the SomaScan assay^18,28^. The abundance of each protein was derived from the relative fluorescence units (RFU) detected from each fluorescent dye. All serum samples were analyzed using the same version of the SomaScan assay and processed in a single batch to ensure consistency and to minimize potential batch effects. To account for sample-specific variability and distributional differences, proteomic data were normalized using adaptive normalization by maximum likelihood.

### Statistical analysis

All statistical calculations were performed using R software (ver. 4.3.2, http://www.r-project.org/). During pre-processing, all RFU values of serum proteins were log_2_-transformed to approximate a normal distribution, and biological sex data were converted to a binary dummy variable (man: 1, woman: 0). The age-adjusted mean difference of log_2_ RFU values in serum protein between men and women was represented by the logarithm of fold change (FC = men/women).

Initially, we checked the quality of the 16-year cryopreserved samples using circulating protein data from the SomaScan assay and from our previous studies reporting serum adiponectin and plasma resistin concentrations^23,24^. Specifically, we evaluated the sample stability by comparing the concentrations of two proteins—adiponectin (measured by ELISA in serum) and resistin (measured by ELISA in plasma)—originally quantified from peripheral blood collected from the same individuals, with those measured 16 years later using SomaScan. Importantly, the blood samples themselves were identical; only the storage duration and measurement methods differed. We calculated Spearman’s rank correlation coefficients to evaluate the correlation between SomaScan assay data in 2023 and those from previous immunoassay measurements.

We then performed a multiple linear regression analysis to assess age-adjusted sex differences in serum protein concentrations measured by the SomaScan assay. This linear regression model included each log_2_ RFU value of serum protein as a dependent variable (Y), and biological sex and age as independent variables (X). *P*-values were adjusted using the Benjamini–Hochberg method^29^setting the significance threshold at a false discovery rate (FDR) = 0.05. We also evaluated the association between age and sex-differential serum protein concentrations detected by the abovementioned analysis. For each group of men and women, a simple linear regression analysis was performed with each log_2_ RFU value as Y and age as X, to assess whether the serum protein concentrations changed with age as a continuous variable at the significance level (α) = 0.05.

## Results

### Sample quality evaluation: Correlation between SomaScan assay and previous measurements of adiponectin and resistin

**Fig. 1:**
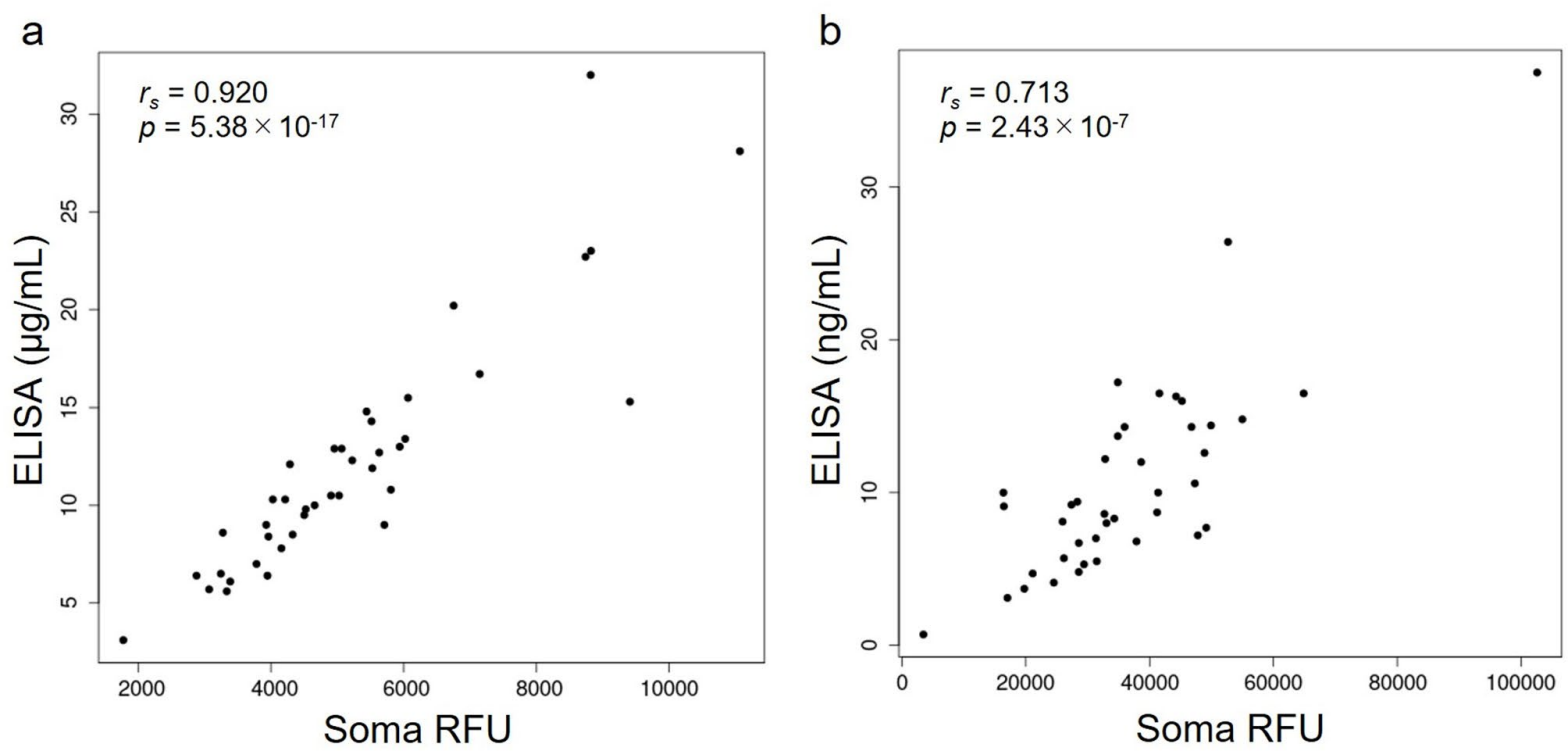
(**a**) Correlation between serum adiponectin concentrations measured by the SomaScan assay and enzyme-linked immunosorbent assay (ELISA; Spearman’s correlation coefficient [*r*_*s*_] = 0.920, *p* = 5.38 × 10^− 17^). (**b**) Correlation between serum resistin concentrations measured by the SomaScan assay and plasma resistin concentration measured by ELISA (*r*_*s*_ = 0.713, *p* = 2.43 × 10^− 7^).

The SomaScan assay was conducted according to the standardized quality control processes (e.g., calibration and normalization) of SomaLogic Inc., with the remaining 7,596 measured proteins. Of these, 307 proteins not of human origin were not included in our analysis, and a total of 7,289 protein data points were included. Previous immunoassay data were combined with the SomaScan assay dataset, and the correlation between the previous immunoassays of adiponectin and resistin and the SomaScan assay measurements was obtained (Fig. 1). Serum concentrations of adiponectin in the same individuals at different measurement time points and assay methods were highly correlated (*r*_*s*_ = 0.920, *p* = 5.38 × 10^− 17^). Despite the differences between the serum and plasma samples, the serum resistin and plasma resistin measurements using the SomaScan assay and ELISA were also highly correlated (*r*_*s*_ = 0.713, *p* = 2.43 × 10^− 7^).

### Sex differences in serum protein concentrations

**Fig. 2:**
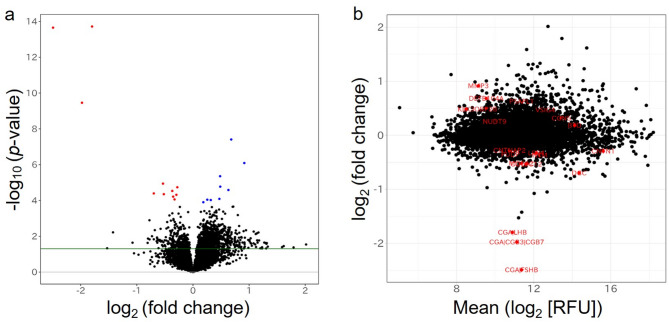
(**a**) Volcano plot differentiating serum protein concentrations between men and women. Blue and red dots represent concentrations significantly higher in men and women, respectively. The horizontal green line indicates *p*-value = 0.05. (**b**) MA plot illustrating the association between serum protein concentrations and sex. Serum protein concentrations and sex represent the logarithm of fold change and the mean logarithm of relative fluorescence units.

In the age-adjusted linear regression models, we evaluated the sex differences in serum protein concentrations (see **Additional file 1** for details), and created volcano and MA plots of the results (Fig. 2**)**. We found 20 serum proteins with significant sex differences (FDR < 0.05): nine of these were higher in men, and 11 were higher in women (Fig. 2a). These significant serum proteins were not at extremely high or low concentration ranges compared to all serum proteins (Fig. 2b). Of the significant serum proteins, the top three with the greatest sex differences were heterodimers: glycoprotein hormone α chain/follitropin subunit β (CGA|FSHB), glycoprotein hormone α chain/choriogonadotropin subunit β3/choriogonadotropin subunit β7 (CGA|CGB3|CGB7), and glycoprotein hormone α chain/lutropin subunit β (CGA|LHB). The serum concentrations of this gonadotropic hormone were more than twofold higher in women than in men (log_2_ (FC) < -1.0, Table 1). However, the RFU measurements of each β subunit of gonadotropic hormone did not differ between sexes (Table 1) when SOMAmer, which can recognize only the β subunit, was used.

**Table 1:** Summary of serum protein concentrations that were significantly differed between men and women.

Sequence ID	Full Name	Entrez Gene Name	log_2_FC	SE	*p*-value	*q*-value
Higher serum concentration in men						
5763-67	Beta-defensin 104	DEFB104A	0.68	0.10	3.92 × 10^− 8^	7.14 × 10^− 5^
2788-55	Stromelysin-1	MMP3	0.91	0.15	8.09 × 10^− 7^	1.18 × 10^− 3^
4330-4	Prostate-specific antigen	KLK3	0.48	0.09	4.43 × 10^− 6^	5.38 × 10^− 3^
5680-54	Odorant-binding protein 2b	OBP2B	0.49	0.10	1.69 × 10^− 5^	1.49 × 10^− 2^
8814-33	Proactivator polypeptide-like 1	PSAPL1	0.63	0.13	2.61 × 10^− 5^	1.90 × 10^− 2^
17678-28	V-set and immunoglobulin domain-containing protein 4	VSIG4	0.47	0.11	8.15 × 10^− 5^	3.64 × 10^− 2^
9482-110	ADP-ribose pyrophosphatase, mitochondrial	NUDT9	0.26	0.06	9.06 × 10^− 5^	3.64 × 10^− 2^
6392-7	WNT1-inducible-signaling pathway protein 2	CCN5	0.32	0.07	9.50 × 10^− 5^	3.64 × 10^− 2^
9269-7	Biotinidase	BTD	0.19	0.04	1.25 × 10^− 4^	4.56 × 10^− 2^
Higher serum concentration in women						
2953-31	Luteinizing hormone	CGA|LHB	-1.80	0.15	1.92 × 10^− 14^	8.10 × 10^− 11^
14056-4	Subunit: Glycoprotein hormones alpha chain	CGA	0.00	0.05	0.96	0.98
8376-25	Subunit: Lutropin subunit beta	LHB	-0.13	0.08	0.10	0.38
3032-11	Follicle-stimulating hormone	CGA|FSHB	-2.49	0.21	2.22 × 10^− 14^	8.10 × 10^− 11^
8036-75	Subunit: Follitropin subunit beta	FSHB	0.26	0.11	0.02	0.23
4914-10	Human Chorionic Gonadotropin	CGA|CGB3|CGB7	-1.98	0.23	3.48 × 10^− 10^	8.46 × 10^− 7^
21391-17	Subunit: Choriogonadotropin subunit beta 3	CGB3	0.10	0.08	0.22	0.52
5457-5	Collectin-12	COLEC12	-0.53	0.11	1.15 × 10^− 5^	1.19 × 10^− 2^
6965-19	Contactin-associated protein-like 2	CNTNAP2	-0.28	0.06	1.83 × 10^− 5^	1.49 × 10^− 2^
8428-102	Neurotrimin	NTM	-0.37	0.08	2.95 × 10^− 5^	1.95 × 10^− 2^
21685-29	DCC	DCC	-0.70	0.15	4.04 × 10^− 5^	2.46 × 10^− 2^
19372-7	MAM domain-containing glycosylphosphatidylinositol anchor protein 2	MDGA2	-0.52	0.11	4.45 × 10^− 5^	2.50 × 10^− 2^
2974-61	Contactin-1	CNTN1	-0.30	0.06	4.81 × 10^− 5^	2.51 × 10^− 2^
20912-10	Pseudouridine-5’-phosphatase	PUDP	-0.35	0.08	6.12 × 10^− 5^	2.97 × 10^− 2^
19558-10	Low-density lipoprotein receptor-related protein 4	LRP4	-0.33	0.07	8.72 × 10^− 5^	3.64 × 10^− 2^

Logarithm of fold change (log_2_FC, men/women) representing the difference in serum protein concentration between 20 male and 20 female participants. Age-adjusted linear regression model was used to calculate *p*-values, which were adjusted using the Benjamini–Hochberg method as the *q*-values, setting the significance threshold for the false discovery rate (FDR) of 0.05.

Abbreviation: SE, standard error.

However, sex differences in serum concentrations of the nine proteins with higher serum concentrations in men than in women were all less than two-fold (Table 1); the top three serum proteins with higher concentrations in men were β-defensin 104 (DEFB104A), matrix metalloproteinase-3/stromelysin-1 (MMP3), and prostate-specific antigen/kallikrein-related peptidase 3 (PSA/KLK3).

In each male and female group, the 11 and nine serum proteins with sex differences were evaluated to determine whether these were associated with age (Table 2). No serum protein concentration with a significant sex difference was associated with age, except neurotrimin (NTM, *p* = 0.039) and V-set immunoglobulin domain-containing protein 4 (VSIG4, *p* = 0.020).

**Table 2:** Association between age and each serum protein concentration showing significant differences by sex.

			Men				Women		
Sequence ID	Full Name	Entrez Gene Name	β	SE	*p*-value		β	SE	*p*-value
2953-31	Luteinizing hormone	CGA|LHB	0.006	0.016	0.706		-0.006	0.018	0.736
3032-11	Follicle stimulating hormone	CGA|FSHB	0.003	0.029	0.908		0.023	0.016	0.163
4914-10	Human Chorionic Gonadotropin	CGA|CGB3|CGB7	-0.005	0.023	0.840		-0.014	0.030	0.646
5763-67	Beta-defensin 104	DEFB104A	-0.004	0.015	0.776		-0.006	0.004	0.163
2788-55	Stromelysin-1	MMP3	0.007	0.016	0.674		-0.020	0.018	0.283
4330-4	Prostate-specific antigen	KLK3	0.010	0.013	0.464		0.002	0.007	0.823
5457-5	Collectin-12	COLEC12	0.015	0.010	0.181		0.004	0.013	0.781
5680-54	Odorant-binding protein 2b	OBP2B	0.011	0.014	0.450		-0.006	0.006	0.345
6965-19	Contactin-associated protein-like 2	CNTNAP2	0.009	0.007	0.214		0.005	0.006	0.453
8814-33	Proactivator polypeptide-like 1	PSAPL1	0.009	0.012	0.485		-0.021	0.017	0.216
8428-102	Neurotrimin	NTM	0.016	0.007	0.039		0.009	0.010	0.383
21685-29	DCC	DCC	0.017	0.014	0.217		0.016	0.020	0.433
19372-7	MAM domain-containing glycosylphosphatidylinositol anchor protein 2	MDGA2	0.010	0.012	0.446		0.002	0.013	0.873
2974-61	Contactin-1	CNTN1	0.008	0.007	0.281		0.009	0.008	0.258
20912-10	Pseudouridine-5’-phosphatase	PUDP	0.012	0.007	0.104		0.019	0.010	0.084
17678-28	V-set and immunoglobulin domain-containing protein 4	VSIG4	0.017	0.015	0.256		0.023	0.009	0.020
6392-7	WNT1-inducible-signaling pathway protein 2	CCN5	0.000	0.008	0.984		-0.007	0.009	0.423
19558-10	Low-density lipoprotein receptor-related protein 4	LRP4	0.016	0.008	0.065		0.010	0.008	0.238
9482-110	ADP-ribose pyrophosphatase, mitochondrial	NUDT9	-0.003	0.006	0.667		0.000	0.007	0.998
9269-7	Biotinidase	BTD	-0.007	0.004	0.092		-0.004	0.006	0.484

The simple linear regression was used to calculate the standardized coefficient (β) and standard error (SE) in male and female groups.

## Discussion

The present study investigated the possibility of using long-term cryopreserved serum samples with proteomics techniques and sex differences in circulating proteins in East Asians using their long-term cryopreserved samples. In the sample quality evaluation, we used ELISA-measured concentrations of adiponectin and resistin, both of which have been previously reported to be associated with their respective gene polymorphisms^23,24^. The serum concentration of adiponectin measured previously^23^ was almost identical to that measured by the SomaScan assay in the present study (*r*_*s*_ = 0.920, *p* = 5.38 × 10^− 17^). In the evaluation of resistin measurements, a certain degree of correlation was confirmed between previous measurements^24^ and those using the SomaScan assay, although the correlation was not as high as that observed with adiponectin. One reason for the difference in this correlation is that resistin measurements were conducted using plasma samples in previous studies, whereas adiponectin measurements were made using serum samples. Given the different protein signatures of plasma and serum^20^it is possible that our long-term cryopreserved samples experienced minimal degradation and retained the potential to analyze their partial proteomic information. Our study presents evidence that long-term cryopreserved serum samples can at least be used in future studies of adiponectin, which is closely related to the pathophysiology of diabetes^30^cardiovascular disease^31^other metabolic diseases^32^.

Considering these results, a proteome analysis was performed using the long-term stored cryopreservation samples of the same 40 elderly Japanese participants, to evaluate sex differences in the serum protein concentrations. In women, serum concentrations of CGA|FSHB, CGA|CGB3|CGB7, and CGA|LHB (i.e., gonadotropic hormones) were more than two-fold higher than those of men, and these results were consistent with those of a large study involving other ethnic groups^19^. While menopausal status was not directly assessed in this study, the observed sex differences in gonadotropin levels may reflect expected physiological differences in older adults. Notably, regression analyses did not show significant age-related variation within this age composition, which aligns with prior literature suggesting that gonadotropin concentrations stabilize several years after menopause^33–36^. However, as menopausal status was not documented, these interpretations should be made with caution. Furthermore, the SomaScan platform measures heterodimeric forms of gonadotropins such as CGA|FSHB, CGA|CGB3|CGB7, and CGA|LHB, which represent the biologically active forms of these hormones. This contrasts with assays that detect individual subunits and may not reflect functional hormone concentrations. The ability to quantify intact heterodimers enhances the interpretability of proteomic data and highlights the utility of SomaScan for investigating physiologically relevant hormonal patterns. However, there were no differences between the serum concentrations of subunits of these heterodimer gonadotropins between men and women using the SomaScan assay. Therefore, the feasibility of measuring solely subunits in long-term cryopreserved serum samples using the SomaScan assay requires further investigation. In addition, our study showed that serum levels of DEFB104A, MMP3, and PSA/KLK3 were higher in males than in females. According to the Human Protein Atlas (Version: 24.0; https://www.proteinatlas.org/), both DEFB104A and PSA/KLK3 are highly expressed in male-specific organs such as the epididymis and prostate. Additionally, higher serum concentrations of MMP-3 have been reported in men than in women in healthy populations^37^which is consistent with our present findings.

Furthermore, we identified NTM and VSIG4 as circulating proteins whose serum concentrations increase with age in men and women, respectively. NTM is a cell adhesion molecule closely related to neural connectivity and neurite outgrowth, and it is also expressed at high levels primarily in the olfactory bulb, neural retina, dorsal root ganglia, and spinal cord^38^. Recent genome-wide association studies have reported that four SNPs located within intron 1 of the *NTM* gene are associated with late-onset Alzheimer’s disease^39^. VSIG4 is highly expressed on tissue resting macrophages, the giant red pulp progenitor cells derived from fetal liver Kupffer cells, and is closely related to opsonophagocytosis of pathogens and the subsequent inflammatory response process^40–42^. VSIG4 is associated with insulin resistance and the epithelial-mesenchymal transition of kidney tubular cells under hyperglycemia^43,44^. Although our proteomic study was small-scale, it provided important insights into differences between the sexes with age in East Asian populations, particularly regarding neurological and immunological aspects.

However, our study has several limitations: first, the sample quality evaluation was performed only for resistin and adiponectin. Thus, we should be cautious when evaluating other serum proteins outside the nanogram to microgram per milliliter order range of the easily catabolized magnitude, especially those present in trace amounts. Second, the effects of remelting and refreezing were not considered in the possibility of using the long-term cryopreserved samples presented in this study. To quantitatively assess time-dependent serum protein degradation and the impact of repeated freeze–thaw cycles, future longitudinal studies using samples stored for varying durations are necessary. Third, in the exploratory analyses assessing the relationship between age and serum protein levels that showed sex differences, statistical significance was evaluated based only on nominal *p*-values without adjustment for multiple testing. As such, these results should be interpreted with caution and cannot support definitive conclusions. Finally, the sample size of this study was relatively small, and there was no validation dataset. To test the robustness of our results, it is necessary to apply them to another long-term sample from a population different from that in the present study. Consistency with another assay method also needs to be confirmed in the future.

## Conclusions

Despite the limitations, the present study is the first to report the possibility of using long-term cryopreserved serum samples in the SomaScan assay, a novel proteomics platform. Many biobanks have stored numerous biospecimens; therefore, our results could contribute to the successful utilization of their sample properties. In addition, we identified heterodimer gonadotropic hormones as protein markers that were present at more than twice the serum concentration in elderly Japanese women compared with that in elderly Japanese men.

## Supplementary Information

Below is the link to the electronic supplementary material.

Supplementary Material 1

## Data Availability

Availability of Data and Materials: All data supporting the conclusions of this article are included within the article.
